# Disease progression patterns and molecular resistance mechanisms to crizotinib of lung adenocarcinoma harboring ROS1 rearrangements

**DOI:** 10.1038/s41698-022-00264-w

**Published:** 2022-03-31

**Authors:** Yongchang Zhang, Zhe Huang, Liang Zeng, Xiangyu Zhang, Yizhi Li, Qinqin Xu, Haiyan Yang, Analyn Lizaso, Chunwei Xu, Jun Liu, Wenxian Wang, Zhengbo Song, Sai-Hong Ignatius Ou, Nong Yang

**Affiliations:** 1 Department of Medical Oncology, Lung Cancer and Gastrointestinal Unit, Hunan Cancer Hospital/The Affiliated Cancer Hospital of Xiangya School of Medicine, Central South University, 410013 Changsha, China; 2 Graduate Collaborative Training Base of Hunan Cancer Hospital, Hengyang Medical School, University of South China, Hengyang, 421001 Hunan China; 3 Department of Medical Oncology, Qinghai Provincial People’s Hospital, 810000 Xining, China; 4 Burning Rock Biotech, 510300 Guangzhou, China; 5 Department of Respiratory Medicine, Jinling Hospital, Nanjing University School of Medicine, Nanjing, China; 6 Cancer Center, Union Hospital, Tongji Medical College, Huazhong University of Science and Technology, 430022 Wuhan, China; 7 Department of Medical Oncology, Cancer Hospital of the University of Chinese Academy of Sciences (Zhejiang Cancer Hospital), Hangzhou, 310022 Zhejiang China; 8 Chao Family Comprehensive Cancer Center, Department of Medicine, Division of Hematology-Oncology, University of California Irvine School of Medicine, Orange, CA USA

**Keywords:** Lung cancer, Predictive markers

## Abstract

This retrospective study investigated the association between the pattern of disease progression and molecular mechanism of acquired resistance in a large cohort of 49 patients with *ROS1*-rearranged advanced non-small-cell lung cancer treated with first-line crizotinib. We found that treatment-emergent *ROS1* point mutations were the major molecular mechanism of crizotinib resistance, particularly for patients who developed extracranial-only disease progression. Our findings highlight the importance of rebiopsy and gene testing for subsequent-line therapeutic management.

Gene rearrangements involving the ROS proto-oncogene-1 (ROS1) are actionable therapeutic targets for non-small cell lung cancer (NSCLC). *ROS1* fusions occur at a rate of 2% in NSCLC and up to 3.3% in lung adenocarcinoma^1,2^. Crizotinib is the first tyrosine kinase inhibitor (TKI) to show clinical activity in *ROS1*-rearranged NSCLC^3^. Cancer cells that are previously sensitive to TKIs such as crizotinib develop resistance thru on-target and off-target mechanisms, which are the main causes of treatment failure and lead to disease progression^4^. Brain metastasis is commonly observed in patients with lung cancer and is also one of the primary modes of disease progression in *ROS1*-positive patients^5^. So far, few studies have comprehensively analyzed the frequency of *ROS1* resistance mutations and the pattern of disease progression after crizotinib therapy of *ROS1*-rearranged NSCLC^6^. This retrospective study aimed to explore the molecular mechanism of crizotinib resistance and its relationship with the mode of progression.

Of the 117 patients screened, 49 patients with *ROS1*-rearranged advanced lung adenocarcinoma were included in our analysis. A total of 30 patients (61.2%, 30/49) were identified with secondary *ROS1* mutations using NGS analysis of rebiopsy samples collected at progression from first-line crizotinib. The study cohort comprised of 57.1% female (*n* = 28), 75.5% patients who had no smoking history (*n* = 37), and 67.3% of patients without baseline brain metastasis (*n* = 33). The median age of 50 years (range: 26–66). All patients had lung adenocarcinoma (100%). *CD74*-*ROS1* was observed in 57.1% (28/49) of the cohort and was the most common fusion partner of *ROS1*. *SDC4-ROS1* was detected in ten patients (20.4%, 10/49), *EZR-ROS1* in six patients (12.2%, 6/49), two patients each (4,1%, 2/49) with *SLC34A2-ROS1* and *TPM3-ROS1*, and a patient (2%, 2.1/49) had *CCDC6-ROS1*. Most patients (91.8%, 45/49) had single gene fusion, whereas the remaining four patients (8.2%, 4/49) had multiple gene fusions wherein one gene fusion is a canonical *ROS1* gene fusion, and the other is a retained 5′-*ROS1* fused to another gene. Table 1 lists the baseline characteristics of the cohort.

**Table 1:** Baseline characteristics of 49 Crizotinib-treated patients with advanced NSCLC who provided rebiopsy samples for NGS detection at crizotinib progression.

Characteristics	*n* (%)
Age at diagnosis (years), median (range)	50 (26–66)
Sex	
Male	21 (42.9)
Female	28 (57.1)
Smoking history	
With smoking history	12 (24.5)
Without smoking history	37 (75.5)
Histology	
Adenocarcinoma	49 (100)
Squamous cell carcinoma	0 (0)
Brain metastasis status at baseline	
With	16 (32.7)
Without	33 (67.3)
ROS1 fusion partner	
CD74-ROS1	28 (57.1)
SDC4-ROS1	10 (20.4)
EZR-ROS1	6 (12.2)
SLC34A2-ROS1	2 (4.1)
TPM3-ROS1	2 (4.1)
CCDC6-ROS1	1 (2.1)
CD74-ROS1 + ROS1-MRAS*	1 (2.1)
CD74-ROS1 + ROS1-PUM1*	1 (2.1)
EZR-ROS1 + ROS1-BTBD9*	1 (2.1)
CD74-ROS1 + ROS1-HMGXB3*	1 (2.1)

Asterisk (*) denotes previously unreported *ROS1* fusions.

For patterns of disease progression analyses, all 49 patients were included. Patient details are listed in Supplementary Table 1. Extracranial-only progression was confirmed in 33 (67.3%) patients, intracranial-only progression in 11 (22.4%) patients, and both intracranial and extracranial progression in five (10.2%) patients (Fig. 1). The clinical characteristics among the three groups were not statistically different (Supplementary Table 2). The detailed information of the patients with intracranial-only progression and both intracranial and extracranial progression are also summarized in Supplementary Table 3 and Supplementary Table 4, respectively.

**Fig. 1:**
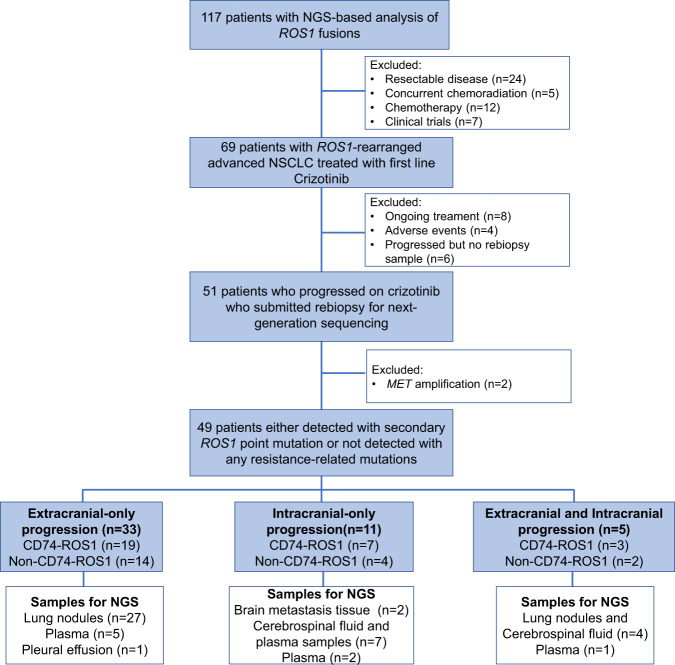
Study profile. Flow chart illustrating the study design.

Treatment-emergent *ROS1* point mutations were detected in 61.2% of patients (30/49) after crizotinib progression, with *ROS1* G2032R being the most common mutation, detected in 14 patients (28.5%, 14/49). The other secondary *ROS1* mutations detected in our cohort were G2032K (8.3%, 4/49), G2026M (6.1%, 3/49), L2086F (6.1%, 3/49), S1986Y (4.1%, 2/49), S1986F (2%, 1/49), L1174F (2%, 1/49), and L2155S (2%, 1/49). The remaining 19 (38.7%, 19/49) patients were not detected with any mutations related to crizotinib resistance (Fig. 2a).

**Fig. 2:**
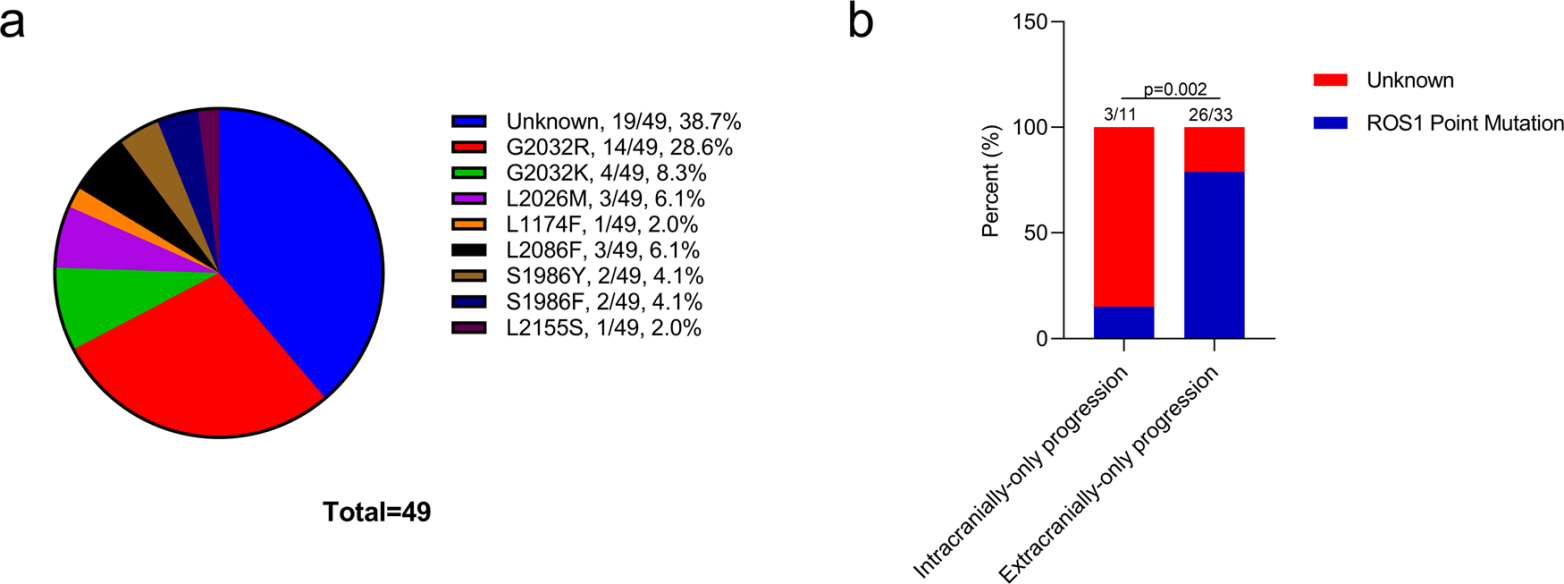
Patterns of disease progression and molecular mechanisms of resistance. **a** Distribution of molecular mechanisms of crizotinib resistance of the cohort. **b** Patients with intracranial-only progression on crizotinib had a significantly lower frequency of treatment-emergent *ROS1* point mutations than patients with extracranial-only progression.

Of the 11 patients with intracranial-only progression, the sample types submitted for NGS testing were cerebrospinal fluid (positive cytology, as liquid biopsy sample) and plasma samples (*n* = 7), plasma samples (*n* = 2), or brain metastasis tissue sample (*n* = 2) (Fig. 1). We further compared the frequency of treatment-emergent *ROS1* point mutations in patients with either intracranial or extracranial progression. Comparative analysis revealed that patients with extracranial-only progression had a significantly higher frequency of *ROS1* point mutations than patients with intracranial-only progression (72.7% vs. 15.2%, *p* = 0.001, Fig. 2b). No significant difference was observed between the patients with *CD74-ROS1* and non-*CD74 ROS1* fusions for the frequency of treatment-emergent *ROS1* mutations (70.4% vs. 52.9%, *p* = 0.337, Supplementary Fig. 1) and the rates of intracranial or extracranial progression (57.63% vs. 63.6%, *p* = 1.000, Supplementary Fig. 2).

Supplementary Table 4 summarizes the clinical information of the five patients with both intracranial and extracranial metastasis at crizotinib progression. These five patients were comprised of two patients harboring *CD74-ROS1*, two patients with *SDC4-ROS1*, and a patient with *TPM3-ROS1*. A patient was detected with *ROS1* L1174F, while no mutation was detected in the other four patients.

The molecular mechanisms of acquired resistance to crizotinib have been well-described both in vitro and in patients with *ROS1*-rearranged lung cancer^7–10^. Previous studies have shown that the mechanisms of crizotinib resistance in patients with *ROS1* fusion-positive NSCLC are mainly divided into two categories: on-target mechanisms involving mutations in the ROS1 kinase domain and off-target mechanisms involving the activation of other signaling pathways^11–14^. However, most of these clinical studies reporting on the inhibitor resistance mechanisms of *ROS1*-rearranged lung cancer were conducted with only limited samples, and only a few explored the association between treatment-emergent *ROS1* point mutations and patterns of disease progression. Hence, our study extends the understanding of this association by performing retrospective analysis of a larger cohort of patients with *ROS1*-rearranged advanced NSCLC treated with crizotinib.

In our cohort, 57.4% had *CD74-ROS1* at diagnosis, which is consistent with previous reports^10,15^. We also found four patients with multiple fusions, which may pose as a predictive factor for the poor prognosis of patients harboring *ROS1* fusions^16^. Although these 5’-*ROS1* fusions are not expressed into RNA and protein, and no study has provided evidence on their oncogenic function, we speculate that the detection of these 5’-end fusions using DNA-NGS reflects a more complex biological scenario^17^. Future studies are warranted to shed light on the molecular mechanisms associated with the retention of these 5′-*ROS1* fusions.

Among the patients in our cohort, ten patients had brain progression. Approximately 63.8% of patients were detected with treatment-emergent *ROS1* point mutations after crizotinib progression and were not detected at baseline. *ROS1* G2032R was the most common, which is consistent with other published reports^1,10,15,18^. The secondary *ROS1* missense mutations detected from our cohort were all previously reported. These *ROS1* missense mutations affect the residues located in the ATP binding pocket of the kinase domain, resulting in steric hindrance and blocking the binding of ROS1 inhibitors at varied levels, which leads to inhibitor resistance^1,10,15,18^. Our results support that secondary mutations in the ROS1 kinase domain are the major molecular mechanisms of crizotinib resistance in patients with *ROS1*-rearranged NSCLC, particularly those with extracranial progression. After crizotinib progression, these patients with secondary mutations could benefit from next-generation ROS1 inhibitors such as entrectinib, lorlatinib, or cabozantinib, depending on the sensitivity profile of the *ROS1* point mutation^15^. This raises the need to perform rebiopsy and elucidate the mutational profile after developing disease progression to understand the resistance mechanism and plan optimal therapeutic strategies to improve the survival outcomes of these patients. In addition, no resistance-related mutation was detected in some patients, particularly those with intracranial progression, despite the use of their cerebrospinal fluid or brain tissue samples for molecular analysis, suggesting that unknown resistance mechanism needs further exploration. As previously discussed by Gainor et al., patients with intracranial progression might reflect pharmacokinetic failure due to the inherent limitation of crizotinib in penetrating the blood-brain barrier, rather than true biological resistance^10^. Moreover, the comparable modes of disease progression and rates of secondary *ROS1* point mutations in patients with either *CD74-ROS1* or non-*CD74-ROS1* suggest that the *ROS1* gene fusion partners did not contribute to the differences in either the site of disease progression or the mechanisms of acquired resistance.

Our study is limited by its retrospective nature. Some data were not available for analysis and our study only included a small cohort of patients treated in our institution, which may introduce sample bias. Since the cohort with survival outcomes was based on the patients who submitted rebiopsy samples for NGS, inherent sampling bias might exist. Our study only included the analysis of treatment-emergent *ROS1* point mutations as the acquired resistance mechanism. Other *ROS1*-independent mechanisms of resistance should be investigated further. Multi-omics analysis would also be an interesting avenue to comprehensively understand transcriptomics, proteomics, or metabolomics-related changes at crizotinib progression.

In conclusion, our study provides real-world clinical evidence of the association between the molecular mechanisms of resistance and patterns of disease progression in patients with *ROS1*-rearranged advanced NSCLC who received first-line crizotinib. Our findings revealed that treatment-emergent *ROS1* point mutations were the main mechanism of crizotinib resistance, particularly in patients with extracranial progression. These findings raise the need to develop effective treatment strategies for overcoming crizotinib resistance in patients with *ROS1*-rearranged advanced NSCLC.

## Methods

### Patients

We retrospectively analyzed the NGS data of 117 patients with *ROS1*-rearranged NSCLC. The patients were grouped according to the pattern of disease progression. Group 1 included the patients with intracranial-only disease progression (*n* = 11); group 2 included the patients with extracranial-only disease progression (*n* = 33), and group 3 included the patients with both extracranial and intracranial disease progression (*n* = 5) (Fig. 1). Disease progression is evaluated based on radiographic assessment using computed tomography scanning and magnetic resonance imaging for all patients. All patients provided written informed consent to take part in the study. Approval was obtained from the Hunan Cancer Hospital Institutional Review Board Committee (2017YYQ-SSB-026). All patients provided written informed consent to take part in the study.

### NGS

Patient samples from baseline and at confirmation of disease progression were submitted for NGS-based multi-gene panel mutation analysis to Burning Rock Biotech, a College of American Pathologists (CAP)-accredited, Clinical Laboratory Improvement Amendments-certified clinical laboratory. All the gene panels used in our study interrogated whole exons and critical introns of at least the eight classic NSCLC oncogenic drivers, including *EGFR*, *ALK*, *BRAF*, *ERBB2*, *KRAS*, *MET*, *RET*, and *ROS1*. The sequencing analyses were performed using optimized bioinformatics pipeline for somatic variant calling that involved accurate identification of base substitutions, small insertions-deletions, copy number variations, and genomic rearrangements as described previously^19^.

### Statistical analysis

We analyzed the NGS results and patterns of disease progression for each group. We compared the frequency of treatment-emergent *ROS1* point mutations according to progression pattern and type of *ROS1* fusion using chi-square test. *P* < 0.05 indicated statistical significance. All statistical analyses were performed as two-sided tests using SPSS software (version 22) or GraphPad Prism (version 8).

### Reporting summary

Further information on research design is available in the Nature Research Reporting Summary linked to this article.

## Supplementary information

Supplementary Figure 1-2 and Supplementary Table 1-4

REPORTING SUMMARY

## Data Availability

All the data analyzed for this study are included as Supplementary Table 1.

## Code availability

No custom code or scripts were generated or used in our study.
