# Supplementary material for: Disease progression patterns and molecular resistance mechanisms to crizotinib of lung adenocarcinoma harboring ROS1 rearrangements
Source: NPJ Precis Oncol. 2022 Mar 31;6:20. doi: 10.1038/s41698-022-00264-w (PMC8971474; doi:10.1038/s41698-022-00264-w)
Supplement: Supplementary file 1 — Supplementary Figure 1-2 and Supplementary Table 1-4 [file 41698_2022_264_MOESM1_ESM.pdf]

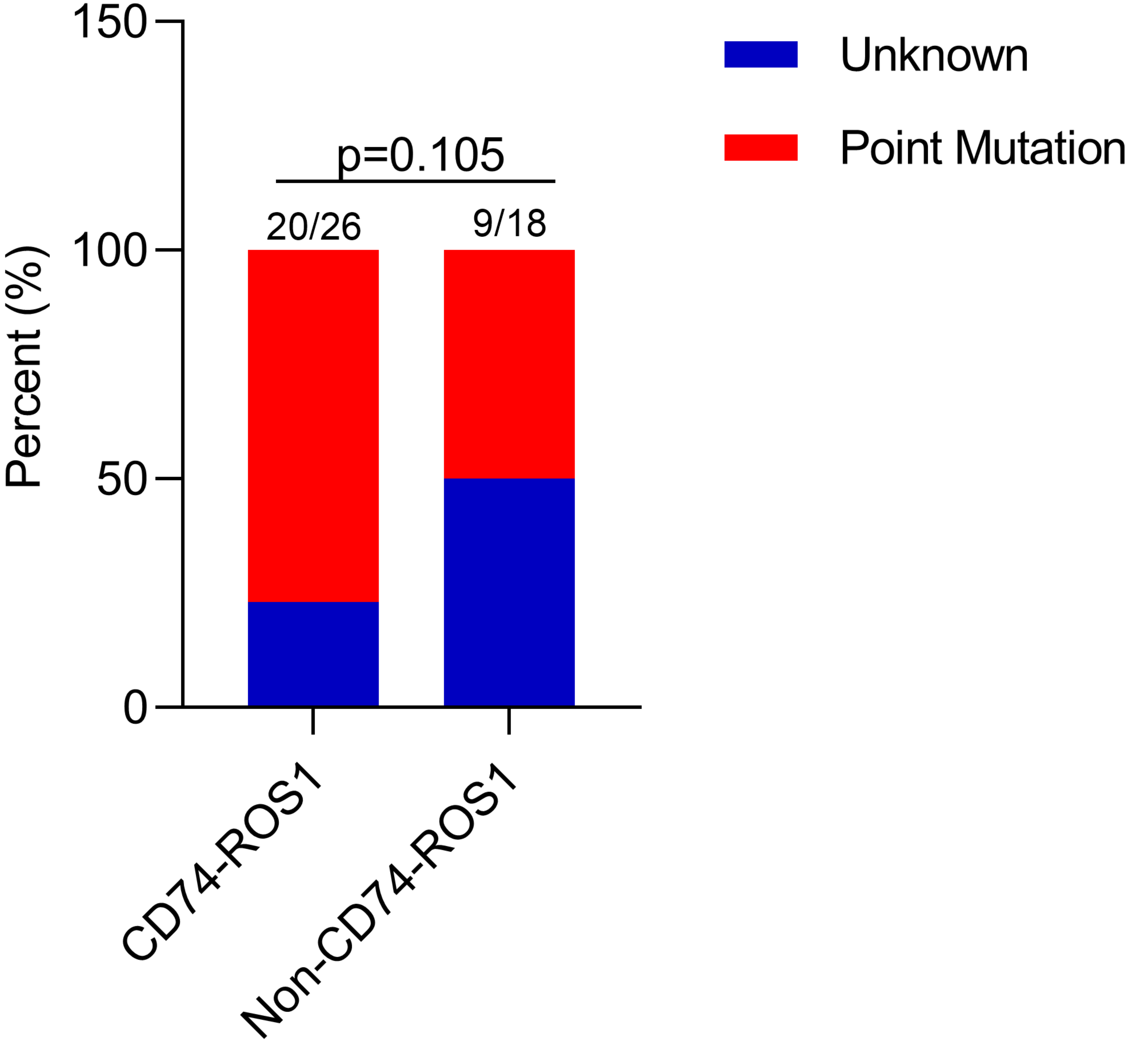

Supplementary Figure 1. Patients with *CD74-ROS1* fusion and non-*CD74-ROS1* fusion had a comparable frequency of treatment-emergent *ROS1* point mutations.

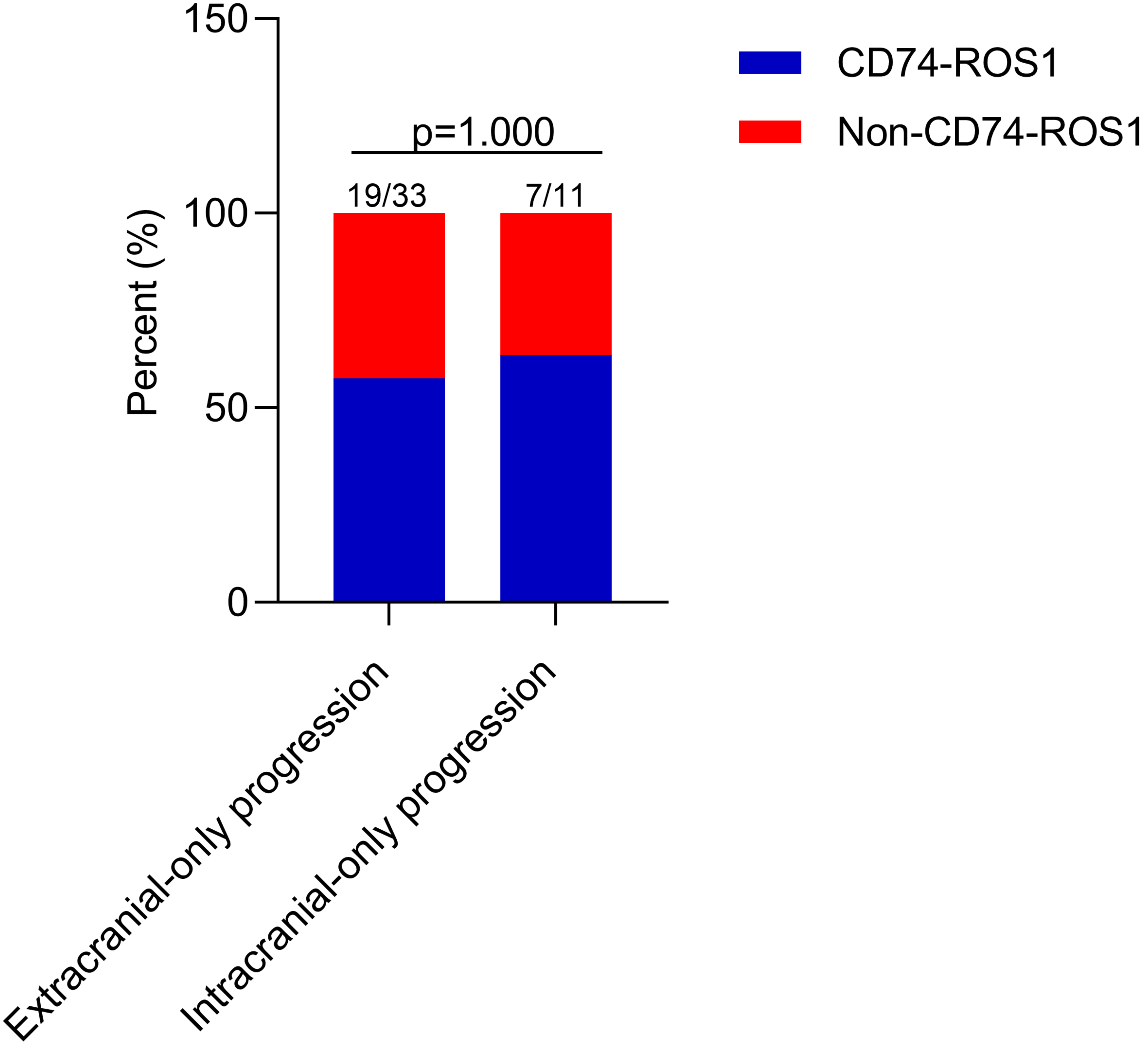

Supplementary Figure 2. Patients with intracranial-only progression and extracranial-only progression had a comparable distribution of *CD74-ROS1* and non-*CD74-ROS1*.

Supplementary Table 1. Detailed clinical and molecular information for all the 49 patients treated with first-line crizotinib who were included in this study.

	Baseline demographics							Clinical outcome with crizotinib	
Patient number	Age	Sex	Smoking history	Tumor histology	Clinical stage	Baseline brain metastasis status	ROS1 fusion detected at baseline by DNA-NGS	Best objective response with crizotinib	PFS on crizotinib therapy (months)
P1	42	Male	No	Adenocarcinoma	IV	No	CD74-ROS1	PR	3
P2	45	Male	Yes	Adenocarcinoma	IV	Yes	SDC4-ROS1	PR	7
P3	65	Male	Yes	Adenocarcinoma	IV	No	CD74-ROS1	SD	5
P4	40	Male	No	Adenocarcinoma	IV	No	CD74-ROS1	PR	27
P5	58	Male	Yes	Adenocarcinoma	IV	Yes	CD74-ROS1	SD	10
P6	51	Female	No	Adenocarcinoma	IV	No	SDC4-ROS1	PR	22
P7	49	Male	No	Adenocarcinoma	IV	No	SDC4-ROS1	PR	15
P8	61	Female	No	Adenocarcinoma	IV	No	CD74-ROS1	PR	13
P9	58	Male	Yes	Adenocarcinoma	IV	Yes	CD74-ROS1+ROS1-MRAS	SD	24
P10	66	Female	No	Adenocarcinoma	IV	No	CCDC6-ROS1	PR	47
P11	49	Female	No	Adenocarcinoma	IV	No	SDC4-ROS1	PR	27
P12	53	Male	No	Adenocarcinoma	IV	No	CD74-ROS1	PR	34
P13	63	Female	No	Adenocarcinoma	IV	No	CD74-ROS1	PR	44
P14	64	Female	No	Adenocarcinoma	IV	No	CD74-ROS1	PR	33.5
P15	57	Male	Yes	Adenocarcinoma	IV	No	SDC4-ROS1	PR	14
P16	47	Female	No	Adenocarcinoma	IV	No	TPM3-ROS1	PR	15
P17	63	Female	No	Adenocarcinoma	IV	Yes	EZR-ROS1	PR	17
P18	56	Male	Yes	Adenocarcinoma	IV	Yes	SDC4-ROS1	PR	12.3
P19	49	Male	Yes	Adenocarcinoma	IV	Yes	EZR-ROS1	PR	13
P20	47	Female	No	Adenocarcinoma	IV	Yes	CD74-ROS1	SD	8
P21	49	Male	No	Adenocarcinoma	IV	No	EZR-ROS1+ROS1-BTBD9	PR	47

Crizotinib progression				
Sites for Progression	Rebiospy sample for NGS at progression	ROS1 fusion detected at progression by DNA-NGS	ROS1 point mutation detected at crizotinib progression and were undetected at baseline	Subsequent treatment after crizotinib resistance
Pleural Metastasis	Pleural effusion	CD74-ROS1	ROS1 G2032R	Chemotherapy
Pleural Metastasis	Plasma	SDC4-ROS1	ROS1 G2032R	Clinical trial
Brain Metastasis	Plasma	CD74-ROS1	none	Chemotherapy
Brain Metastasis	Cerebrospinal fluid and Plasma	CD74-ROS1	ROS1 G2032R	Clinical trial
Leptomeningeal Metastasis	Cerebrospinal fluid and Plasma	CD74-ROS1	none	Clinical trial
Lung nodules enlargement	Lung nodules	SDC4-ROS1	ROS1 G2032K	Clinical trial
Lung new nodules	Plasma	SDC4-ROS1	ROS1 G2032R	Ceritinib
Lung nodules enlargement	Lung nodules	CD74-ROS1	ROS1 L2026M	Chemotherapy
Lung nodules enlargement	Lung nodules	CD74-ROS1+ROS1-MRAS	ROS1 L2026M	Alectinib
Brain Metastasis	Plasma	CCDC6-ROS1	none	Alectinib
Lung nodules enlargement	Lung nodules	SDC4-ROS1	none	Chemotherapy
Lung nodules enlargement	Lung nodules	CD74-ROS1	ROS1 G2032K	Chemotherapy
Lung nodules enlargement	Lung nodules	CD74-ROS1	ROS1 G2032R	Clinical trial
Lung new nodules	Plasma	CD74-ROS1	ROS1 G2032R	Chemotherapy
Lung+Brain Metastasis	Lung nodules and Cerebrospinal fluid	SDC4-ROS1	ROS1 L1174F	Clinical trial
New occurring Lung nodules	Plasma	TPM3-ROS1	none	Chemotherapy
Brain Metastasis	Cerebrospinal fluid and Plasma	EZR-ROS1	none	Ceritinib + Radiotherapy
Lung nodules enlargement	Lung nodules	SDC4-ROS1	ROS1 G2032R	Ceritinib
Lung nodules enlargement	Lung nodules	EZR-ROS1	ROS1 G2032R	Clinical trial
Brain Metastasis	Brain Metastasistissue	CD74-ROS1	ROS1 L2026M	Chemotherapy
Brain Metastasis	Cerebrospinal fluid and Plasma	EZR-ROS1+ROS1-BTBD9	none	Clinical trial

P22	50	Female	No	Adenocarcinoma	IV	Yes	TPM3-ROS1	SD	7
P23	37	Female	No	Adenocarcinoma	IV	No	CD74-ROS1	SD	
P24	58	Female	No	Adenocarcinoma	IIIb	No	CD74-ROS1	SD	28
P25	51	Male	Yes	Adenocarcinoma	IV	Yes	CD74-ROS1	PR	27
P26	42	Female	No	Adenocarcinoma	IV	No	CD74-ROS1	SD	24
P27	50	Female	No	Adenocarcinoma	IV	No	EZR-ROS1	PR	22
P28	46	Male	No	Adenocarcinoma	IV	Yes	CD74-ROS1	PR	10.5
P29	39	Female	No	Adenocarcinoma	IV	No	SDC4-ROS1	PR	10.2
P30	35	Female	No	Adenocarcinoma	IV	No	EZR-ROS1	PR	11.2
P31	53	Female	No	Adenocarcinoma	IV	No	SDC4-ROS1	PR	8.3
P32	33	Male	No	Adenocarcinoma	IV	No	SDC4-ROS1	PR	9.4
P33	58	Female	No	Adenocarcinoma	IV	No	CD74-ROS1	PR	11
P34	49	Female	No	Adenocarcinoma	IV	No	CD74-ROS1	PR	12.2
P35	60	Male	Yes	Adenocarcinoma	IV	Yes	CD74-ROS1	PR	14
P36	54	Male	Yes	Adenocarcinoma	IV	Yes	SLC34A2-ROS1	SD	25
P37	44	Male	Yes	Adenocarcinoma	IV	Yes	CD74-ROS1	SD	22
P38	55	Male	No	Adenocarcinoma	IV	No	EZR-ROS1	PR	37
P39	36	Female	No	Adenocarcinoma	IV	No	CD74-ROS1	PR	30
P40	63	Male	No	Adenocarcinoma	IV	No	CD74-ROS1	PR	16
P41	55	Female	No	Adenocarcinoma	IV	No	CD74-ROS1	PR	7
P42	54	Male	No	Adenocarcinoma	IV	No	SLC34A2-ROS1	PR	10
P43	40	Female	No	Adenocarcinoma	IV	No	CD74-ROS1	PR	11.3
P44	45	Female	Yes	Adenocarcinoma	IV	No	CD74-ROS1	SD	22.5
P45	49	Female	No	Adenocarcinoma	IV	No	CD74-ROS1	PR	30
P46	46	Female	No	Adenocarcinoma	IV	No	SDC4-ROS1	PR	10
P47	26	Female	No	Adenocarcinoma	IV	Yes	CD74-ROS1	PR	24
P48	59	Female	No	Adenocarcinoma	IV	No	CD74-ROS1+ROS1-HMGXB3	SD	23
P49	52	Female	No	Adenocarcinoma	IV	No	CD74-ROS1+ROS1-PUM1	PR	13.3

Lung+Brain Metastasis	Plasma	TPM3-ROS1	none	Ceritinib
Lung new nodules	Plasma	CD74-ROS1	ROS1 L2086F	Chemotherapy
Lung+Brain Metastasis	Lung nodules and Cerebrospinal fluid	CD74-ROS1	none	Clinical trial
Brain Metastasis	Cerebrospinal fluid and Plasma	CD74-ROS1	ROS1 G2032R	Clinical trial
Lung nodules enlargement	Lung nodules	CD74-ROS1	ROS1 S1986Y	Chemotherapy
Lung nodules enlargement	Lung nodules	EZR-ROS1	ROS1 G2032K	Lorlatinib
Lung nodules enlargement	Lung nodules	CD74-ROS1	ROS1 S1986Y	Chemotherapy
Brain Metastasis	Cerebrospinal fluid and Plasma	SDC4-ROS1	none	Clinical trial
Lung nodules enlargement	Lung nodules	EZR-ROS1	ROS1 L2086F	Chemotherapy
Lung nodules enlargement	Lung nodules	SDC4-ROS1	none	Ceritinib
Lung nodules enlargement	Lung nodules	SDC4-ROS1	ROS1 L2086F	Chemotherapy
Lung nodules enlargement	Lung nodules	CD74-ROS1	ROS1 S1986F	Chemotherapy
Lung nodules enlargement	Lung nodules	CD74-ROS1	ROS1 L2155S	Ceritinib
Lung nodules enlargement	Lung nodules	CD74-ROS1	ROS1 G2032R	Chemotherapy
Lung nodules enlargement	Lung nodules	SLC34A2-ROS1	none	Chemotherapy
Lung nodules enlargement	Lung nodules	CD74-ROS1	ROS1 G2032K	Ceritinib
Lung nodules enlargement	Lung nodules	EZR-ROS1	none	Chemotherapy
Lung nodules enlargement	Lung nodules	CD74-ROS1	ROS1 S1986F	Clinical trial
Lung nodules enlargement	Lung nodules	CD74-ROS1	ROS1 G2032R	Chemotherapy
Brain Metastasis	Brain Metastasis tissue	CD74-ROS1	none	Ceritinib
Lung nodules enlargement	Lung nodules	SLC34A2-ROS1	ROS1 G2032R	Chemotherapy
Lung nodules enlargement	Lung nodules	CD74-ROS1	ROS1 G2032R	Clinical trial
Back Metastasis	Lung nodules	CD74-ROS1	none	Ceritinib
Lung+Brain Metastasis	Lung nodules and Cerebrospinal fluid	SDC4-ROS1	none	Clinical trial
Pleural and Brain Metastasis	Lung nodules and Cerebrospinal fluid	CD74-ROS1	none	Clinical trial
Brain Metastasis	Cerebrospinal fluid and Plasma	CD74-ROS1	none	Clinical trial
Lung nodules enlargement	Lung nodules	CD74-ROS1+ROS1-HMGXB3	ROS1 G2032R	Ceritinib
Lung nodules enlargement	Lung nodules	CD74-ROS1+ROS1-PUM1	none	Ceritinib

Supplementary Table 2. Clinical characteristics of the cohort according to mode of disease progression (n=49).

	Extracranial-only progression (n=33)	Intracranial-only progression (n=11)	Extracranial and intracranial progression (n=5)	p value
Age at diagnosis (years), median (range)	54(40-61)	51(26-66)	55(49-58)	0.601
Sex				0.611
Male	15(45.5)	5(45.5)	1(20.0)	
Female	18(54.5)	6(54.5)	4(80.0)	
Smoking history				0.842
Yes	8(24.26)	3(27.3)	4(80)	
No	25(75.8)	8(72.7)	1(20)	
Histology				1.000
Adenocarcinoma	33(100)	11(100)	5(100)	
Squamous cell carcinoma	0(0)	0(0)	0(0)	
ROS1 fusion partner				0.295
CD74-ROS1	19(57.6)	7(63.6)	2(40)	
Non-*CD74-ROS1*	14(42.4)	4(36.4)	3(60)	
Brain metastasis at baseline				0.665
Yes	11(33.3)	4(36.4)	1(20)	
No	22(66.7)	7(63.6)	4(80)	
Radiographic imaging used for baseline assessment				0.115
CT	10(29.4)	1(9.2	2(40)	
MRI	23(70.6)	9(81.2)	3(60)	
PET-CT	0(0)	1(9.2)	0(0)	
Radiographic imaging used for assessing disease progression				1.000
MRI	33(100)	11(100)	5(100)	
CT	0(0)	1(9.2)	0(0)	
PET-CT	0(0)	1(9.2)	0(0)	
Samples submitted for NGS at crizotinib progression				0.425
Lung nodules/ Brain Metastasis/Cerebrospinal fluid samples	26(78.8)	7(63.6)		
Plasma/ pleural effusion	7(21.2)	4(36.4)		

Abbreviations: CT: computed tomography, MRI: Magnetic resonance imaging, PET-CT: positron emission tomography computed tomography.

Supplementary Table 3. Clinical and molecular details of 11 patients with intracranial-only progression

Patient number	Age	Sex	Smoking history	Tumor histology	Clinical stage	Baseline brain metastasis status	*ROS1* fusion	Rebiospy sample for NGS at progression	Best objective response with crizotinib	PFS on crizotinib therapy (months)	Resistance mechanisms
1	65	Female	No	Adenocarcinoma	IV	No	*CD74-ROS1*	Plasma	SD	5	unknown
2	40	Male	No	Adenocarcinoma	IV	No	*CD74-ROS1*	Cerebrospinal fluid and Plasma	PR	27	*ROS1* G2032R
3	58	Male	Yes	Adenocarcinoma	IV	Yes	*CD74-ROS1*	Cerebrospinal fluid and Plasma	SD	10	unknown
4	66	Female	No	Adenocarcinoma	IV	No	*CCDC6-ROS1*	Plasma	PR	47	unknown
5	63	Female	No	Adenocarcinoma	IV	Yes	*CD74-ROS1*	Cerebrospinal fluid and plasma	PR	17	*ROS1* L2026M
6	47	Female	No	Adenocarcinoma	IV	Yes	*CD74-ROS1*	Brain tissue	SD	8	unknown
7	49	Male	No	Adenocarcinoma	IV	No	*EZR-ROS+BTBD9-ROS1*	Cerebrospinal fluid and plasma	PR	47	unknown
8	51	Male	Yes	Adenocarcinoma	IV	Yes	*CD74-ROS1*	Cerebrospinal fluid and plasma	PR	27	*ROS1* G2032R
9	39	Female	No	Adenocarcinoma	IV	No	*SDC4-ROS1*	Cerebrospinal fluid and plasma	PR	10.2	unknown
10	55	Female	No	Adenocarcinoma	IV	No	*CD74-ROS1*	Brain tissue	PR	7	unknown
11	26	Female	No	Adenocarcinoma	IV	Yes	*SDC4-ROS1*	Cerebrospinal fluid and plasma	PR	24	unknown

Abbreviations: PFS, progression-free survival; PR, partial response; SD, stable disease

Supplementary Table 4. Clinical and molecular details of 5 patients with both extracranial and intracranial progression

Patient number	Age	Sex	Smoking history	Histology	Clinical stage	Baseline brain metastasis status	*ROS1* fusion	Extracranial progression sites	Best objective response with crizotinib	PFS on crizotinib therapy (months)	Rebiopsy samples for NGS at progression	Resistance mechanisms
1	57	Male	Yes	Adenocarcinoma	IV	No	*SDC4-ROS1*	Lung nodules	PR	14	Lung nodules and Cerebrospinal fluid	*ROS1* L1174F
2	50	Female	No	Adenocarcinoma	IV	Yes	*TPM3-ROS1*	Bone and Lung Nodules	SD	7	Plasma	unknown
3	58	Female	No	Adenocarcinoma	IIIb	No	*CD74-ROS1*	Lung Nodules	SD	28	Lung nodules and Cerebrospinal fluid	unknown
4	49	Female	No	Adenocarcinoma	IV	No	*CD74-ROS1*	Pleural metastasis	PR	30	Lung nodules and Cerebrospinal fluid	unknown
5	46	Female	No	Adenocarcinoma	IV	No	*SDC4-ROS1*	Pleural metastasis and Lung Nodules	PR	10	Lung nodules and Cerebrospinal fluid	unknown

Abbreviations: PFS, progression-free survival; PR, partial response; SD, stable disease
